# Reactive Arthritis After COVID-19 Infection

**DOI:** 10.7759/cureus.11761

**Published:** 2020-11-28

**Authors:** Ibtisam Jali

**Affiliations:** 1 Medicine, King Abdulaziz University, Jeddah, SAU

**Keywords:** reactive arthritis, covid-19

## Abstract

Severe acute respiratory syndrome coronavirus 2 (SARS-CoV-2), which causes coronavirus disease 2019 (COVID-19), is a novel virus that results in a variety of clinical manifestations. In this report, I describe an uncommon presentation of reactive arthritis (ReA) following COVID-19. I report the case of a 39-year-old woman who presented with arthritis in the small joints of the hands after recovery from COVID-19 infection. To my knowledge, only four cases involving such a presentation have been reported in the literature so far.

## Introduction

Spondyloarthropathies (SpA) are a heterogeneous group of articular inflammatory diseases that share common clinical manifestations such as inflammatory back pain, asymmetric peripheral arthritis, tenosynovitis, dactylitis, enthesitis, and positive human leukocyte antigen B27 (HLA-B27). SpA include ankylosing spondylitis, psoriatic arthritis, enteropathic arthritis, reactive arthritis (ReA), and undifferentiated SpA [1].

ReA can occur following gastrointestinal (GI) and genitourinary (GU) infections, and most commonly occurs up to several weeks after the infection. The most common organisms associated with ReA include *Chlamydia trachomatis, Salmonella enteritidis, Shigella flexneri, Clostridium difficile, Campylobacter jejuni, Ureaplasma urealyticum, Neisseria gonorrhoeae, *and* Escherichia coli*. ReA can also occur following certain respiratory tract infections such as the one caused by *Chlamydia pneumoniae *[2]*.*

Articular involvement in ReA can be either monoarthritis or oligoarthritis. As with other SpA, ReA has a tendency to affect the lower extremities. ReA can present with enthesitis and dactylitis [3]. Non-steroidal anti-inflammatory drugs (NSAIDs) are used as the initial treatment for ReA [4]. Disease-modifying anti-rheumatic drugs (DMARDs) such as sulfasalazine can also be used [5]. Recently, biologics have also been introduced in the management of ReA [6].

Coronavirus disease 2019 (COVID-19), which is caused by severe acute respiratory syndrome coronavirus 2 (SARS-CoV-2), has a wide range of manifestations ranging from being asymptomatic to presenting with mild fever, cough, sore throat, breathlessness, fatigue, and malaise, and thereafter, to a severe disease that causes acute respiratory distress syndrome and multi-organ dysfunction [7]. In this case report, I discuss an uncommon presentation of ReA following SARS-CoV-2 infection.

## Case presentation

A 39-year-old Saudi Arabian woman, who had no known medical illnesses, presented with the following symptoms: fever, sore throat, fatigue, generalized body ache, and headache. She was diagnosed with COVID-19 based on a positive nasopharyngeal swab test. Her symptoms improved within 10 days. A repeat swab test was performed 14 days later, and the result was negative.

Three weeks after the onset of infection, she suddenly started to experience pain in the distal interphalangeal (DIP) and proximal interphalangeal (PIP) joints of the hands. The pain in the fifth DIP joint of her left hand was associated with redness, swelling, and decreased range of movement. The patient had no morning stiffness, history of trauma, vaginal discharge, dysuria, conjunctivitis, or skin rash. There was no back pain, nail changes, history of psoriasis, or history suggestive of inflammatory bowel disease (IBD). She had no family history of psoriasis, IBD, or SpA. There was no history that was suggestive of connective tissue diseases or sarcoidosis or any history of contact with sick adults or kids. The only contact had been with an adult case of COVID-19. She denied any use of illicit drugs and alcoholic beverages.

A clinical examination revealed that her vital signs were normal. She did not have any skin rashes or redness of the eyes. Examination of the cardiovascular and respiratory systems was normal. Examination of the hand showed tenderness in the PIP joint of the second and third fingers and the DIP joint of the fifth finger of the right hand. She also had tenderness in the second PIP joint and tenderness, swelling, and decreased range of motion in the DIP joint of the left-hand fifth finger.

Complete blood count, blood chemistry, and liver function test results were normal. Hepatitis and human immunodeficiency virus (HIV) screenings were negative. Erythrocyte sedimentation rate (ESR) and C-reactive protein (CRP) were within the normal range. Tests for antinuclear antibody (ANA), rheumatoid factor (RF), and anti-cyclic citrullinated peptide (anti-CCP) antibodies were negative. Hand and chest X-rays were also normal.

The patient was administered celecoxib for two weeks, following which pain and swelling disappeared after several days. On further follow-up, two months after the last dose of NSAIDs, the patient’s condition was found to be normal with no more pain or swelling, and her joint examination was also normal.

## Discussion

I presented a case of ReA post-COVID-19. ReA has many differential diagnoses, with the most common ones being gouty arthritis, septic arthritis, rheumatoid arthritis, and other seronegative SpA [8]. Gout was deemed unlikely in this patient given her age. In addition, DIP joint involvement, negative RF, an improvement with short-course NSAIDs, and the patient becoming asymptomatic two months after the treatment made rheumatoid arthritis unlikely too. The absence of fever and normal inflammatory markers ruled out septic arthritis.

The patient did not require admission, and a repeat swab test result was negative. Three weeks after the onset of infection, she started to develop symptoms of arthritis. A literature review revealed the description of four cases of ReA after COVID-19, which further supported the diagnosis. The four patients were in different age groups and had varied comorbidities, disease courses, as well as presentations in the joints of the upper and lower extremities [9-12].

The first case involved a male patient in his 50s who was admitted with COVID-19 pneumonia. On day 21, he developed acute bilateral arthritis in his ankles, with mild enthesitis in his right Achilles tendon, but without rash, conjunctivitis, or preceding diarrhea or urethritis. Arthrocentesis of his left ankle revealed mild inflammatory fluid without monosodium urate or calcium pyrophosphate crystals. The culture of synovial fluid was negative. Tests for syphilis, HIV, anti-streptolysin O (ASO), mycoplasma, *Chlamydia pneumoniae*, ANA, RF, anti-CCP antibody, and HLA-B27 were negative. NSAIDs and intra-articular corticosteroid injection resulted in an improvement of his condition [9]. The second case was a 37-year-old female who presented for evaluation of acute pain in the right hand 12 days after testing positive for SARS-CoV-2. The patient's blood work was unremarkable for elevations in Lyme serology, ANA, RF, and uric acid. She was treated with diclofenac gel, gabapentin, and oral hydromorphone as needed [10]. The third case was a 73‐year‐old man who was admitted with COVID-19 pneumonia. Eight days after the completion of COVID‐19 treatment, he developed swelling, redness, pain, and tenderness in the left first metatarsophalangeal joint as well as the DIP and PIP joints of both hands. RF and anti-CCP antibody tests were negative and the uric acid level was within the normal range. The patient was treated with NSAIDs [11]. The fourth case was a 47-year-old man who was exposed to COVID-19 cases one week before his presentation and tested positive for SARS-CoV-2. He presented with a three-day history of progressive right knee pain and swelling and pain in his glans penis. Test results for HIV, syphilis, chlamydia, and gonorrhea were negative. Arthrocentesis showed gram stain and cultures were negative; no crystals were seen. He was treated with etoricoxib and was administered intra-articular triamcinolone. The patient was late in the course of COVID-19 when he developed arthritis and balanitis [12].

One limitation of this study is that viral arthritis is also a known entity, and we were unable to completely exclude acute viral arthritis. A viral etiology is responsible for 1% of all cases of acute arthritis worldwide, and parvovirus B19, hepatitis B and C, HIV, and alphaviruses are among the most important causes of virally mediated arthritis. Regarding parvovirus arthritis, the most frequent pattern of arthritis/arthralgia is a symmetrical polyarticular rheumatoid-like pattern. The mean interval between the development of the initial manifestation and the onset of arthritis is four days. Patients can have arthritis/arthralgia, and commonly involved joints are the knee, wrist, hand, ankle, and elbow [13,14]; the pattern described here was notably different as the patient started to have the symptoms after complete resolution of the viral illness and she had an arthritis presentation that was different from the symmetrical polyarticular associated with parvovirus. The patient did not have a history of contact with any sick kids at all as a complete lockdown was in place in our country at that time. The only contact had been with a positive COVID-19 adult case at the workplace.

## Conclusions

We are still in the process of fully discovering the consequences of novel SARS-CoV-2. This report presented a case of ReA after COVID-19 infection. To my knowledge, only four such cases have been reported in the literature so far. Rheumatologists should seriously consider ReA as a possible complication of COVID-19, by taking the onset of arthritis into account and after the exclusion of other differential diagnoses by clinical assessment and appropriate investigations.
